# Prognostic Value of Computed Tomographic Coronary Angiography for Long-Term Major Adverse Cardiac Events after Liver Transplantation

**DOI:** 10.3390/jcm10143132

**Published:** 2021-07-15

**Authors:** Doo-Hwan Kim, Young-Kug Kim, Tae-Yong Ha, Shin Hwang, Wooil Kim, Hyun-Jung Koo, Dong-Hyun Yang, Joon-Won Kang, Sung-Gyu Lee

**Affiliations:** 1 Department of Anesthesiology and Pain Medicine, Asan Medical Center, University of Ulsan College of Medicine, Seoul 05505, Korea; dh_kim@amc.seoul.kr (D.-H.K.); kyk@amc.seoul.kr (Y.-K.K.); 2 Division of Hepatobiliary Surgery and Liver Transplantation, Department of Surgery, Asan Medical Center, University of Ulsan College of Medicine, Seoul 05505, Korea; haty@amc.seoul.kr (T.-Y.H.); shwang@amc.seoul.kr (S.H.); sglee2@amc.seoul.kr (S.-G.L.); 3 Department of Radiology and Research Institute of Radiology, Asan Medical Center, University of Ulsan College of Medicine, Seoul 05505, Korea; darkcrom23@naver.com (W.K.); elfin19@gmail.com (H.-J.K.); donghyun.yang@gmail.com (D.-H.Y.)

**Keywords:** cardiovascular disease, computed tomographic angiography, coronary artery calcium score, coronary vessels, liver transplantations

## Abstract

Computed tomographic coronary angiography (CTCA) has prognostic value for early major adverse cardiac events (MACEs) after liver transplantation. However, the association between CTCA and long-term MACEs in liver transplant (LT) recipients remains unknown. We evaluated the association between CTCA and long-term MACEs within 5 years after living donor liver transplantation (LDLT). A total of 628 LDLT recipients who underwent CTCA were analyzed between 2010 and 2012. MACEs were investigated within 5 years after LDLT. The factors associated with long-term MACEs in transplant recipients were evaluated. Only 48 (7.6%) patients developed MACEs. In the Fine and Gray competing risk regression, a coronary artery calcium score (CACS) of >400 combined with obstructive coronary artery disease (CAD) (subdistribution hazard ratio: 5.01, 95% confidence interval: 2.37–10.58, *p* < 0.001), age (1.05, 1.01–1.10, *p* = 0.018), diabetes mellitus (2.43, 1.37–4.29, *p* = 0.002), dyslipidemia (2.45, 1.23–4.70, *p* = 0.023), and creatinine (1.19, 1.08–1.30, *p* < 0.001) were independently associated with long-term MACEs. CACS (>400) combined with obstructive CAD may be associated with MACEs within 5 years after LDLT, suggesting the importance of preoperative noninvasive CTCA in LT recipients. The evaluation of coronary artery stenosis on CTCA combined with CACS may have a prognostic value for long-term MACEs in LT recipients.

## 1. Introduction

Liver transplant (LT) recipients are at a high risk of developing cardiovascular diseases (CVDs), which have emerged as a leading cause of post-transplant morbidity and mortality [1]. Therefore, identifying the risk factors for CVD is crucial for cardiac risk stratification, and proper preoperative cardiac examinations should be mandated [2,3]. However, several known risk factors for CVD such as hyperlipidemia, hypertension, and diabetes are masked by end-stage liver disease, making it difficult to identify LT recipients who are at the highest risk of CVD [4].

Although a myocardial perfusion scan or a dobutamine stress echocardiography is recommended for LT recipients with several known risk factors for coronary artery diseases (CADs) [5], the usefulness of these examinations is limited because of their relatively low sensitivity in detecting CAD in LT recipients [6]. Given the uncertainty of the recommended cardiac examinations, noninvasive computed tomographic coronary angiography (CTCA) emerged as a highly accurate method for diagnosing CAD, comparable to conventional invasive coronary angiography [7]. CTCA is used for obtaining the coronary artery calcium score (CACS) and coronary angiographic images, which allow for the identification of coronary vessel stenosis, characteristics of the plaques, and quantitative calcium deposits in the coronary arteries [8].

Multiple investigations reported that CTCA is significantly correlated with known cardiovascular risk factors and CADs on coronary angiography in LT recipients [9,10]. Recent studies on CTCA in transplant recipients described the prognostic value of a CACS of >400 and CTCA-diagnosed CAD in early postoperative CVD [2,11]. Although the usefulness and advantages of CTCA combined with CACS are emphasized [8], no previous studies have investigated the association between CTCA in combination with CACS and postoperative CVD after LT. Notably, the existing evidence showing the factors related to long-term CVDs after LT is lacking. This study therefore aimed to identify the factors associated with long-term CVD after LT and to evaluate the association between CTCA in combination with CACS and long-term CVDs.

## 2. Materials and Methods

### 2.1. Participants

**Figure 1:**
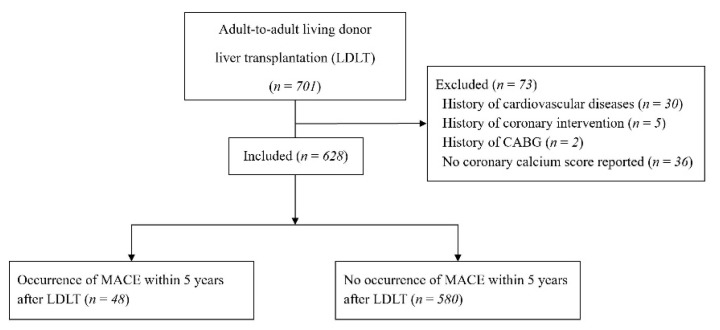
Diagram of the participant selection process. CABG, coronary artery bypass grafting; CTCA, computed tomographic coronary angiography; MACEs, major adverse cardiac events.

This was a single-center, retrospective, observational cohort study of patients who underwent living donor liver transplantation (LDLT) at the Asan Medical Center, Seoul, South Korea and was approved by the Institutional Review Board of Asan Medical Center (2017-0725). All adults (aged ≥ 20 years) who underwent their first LDLT from January 2010 to December 2012 were eligible for inclusion. Patients who (1) underwent orthotopic LT; (2) with no CACS reported; (3) with a preoperative history of CVDs such as stroke, CADs, severe valvular diseases, and significant arrhythmia; and (4) who underwent preoperative coronary intervention or coronary artery bypass graft surgery before LDLT were excluded. In our institution, CTCA has been performed as part of the routine preoperative cardiac evaluation for LT since 2010. Because CTCA was usually conducted in patients scheduled for elective LT, the recipients who underwent orthotopic LT were excluded. Of the 701 LDLT recipients, 36 whose CACS was not reported were excluded. Among the remaining 665 patients, 30 with a preoperative history of CVDs such as stroke, CAD, severe valvular diseases, and significant arrhythmia were excluded. Then, due to the occurrence of symptomatic and severe CAD, 5 patients who underwent coronary intervention and 2 who underwent coronary artery bypass graft surgery before LDLT were also excluded. Finally, 628 patients with no history of chest pain or CVD were included in the analysis (Figure 1).

### 2.2. Routine Preoperative Cardiac Evaluation

In our institution, preoperative cardiac assessments, including an electrocardiogram, echocardiography, and thallium single-photon emission computed tomography (SPECT), were routinely performed. Decreased left-ventricle function, regional wall motion abnormality, significant valvular disease, and pulmonary hypertension were defined as abnormal echocardiographic findings. Meanwhile, a medium or large wall perfusion defect was defined as an abnormal thallium SPECT finding.

### 2.3. Analysis of the CTCA Findings

As part of the routine preoperative cardiac evaluations, CTCA was performed using either a single-source 64 section (VCT XT; General Electric, Milwaukee, WI) or dual-source CT system (Somatom Definition or Somatom Definition Flash, Siemens Medical Solutions, Forchheim, Germany). Coronary artery stenosis was classified as either nonobstructive (<50%) or obstructive (≥50%) stenosis. According to the number of coronary arteries involved, CAD was classified as a 1-, 2-, or 3-vessel disease. Because only 1 patient had 3-vessel disease in our cohort, 3-vessel disease was included in the ≥2-vessel disease category for further analysis. Only 1 patient had left main coronary artery stenosis, which was considered as a 2-vessel disease. Coronary plaques were categorized as calcified (composed exclusively of high-density material, >130 HU), noncalcified (composed exclusively of material having a density of ≤130 HU), and mixed (having components of both calcified and noncalcified plaques) [12]. For analysis of coronary plaques, in case several plaque types were present, the characteristics of the severely stenotic plaque were selected for statistical analysis. CTCA also provides another important value, the CACS, which denotes the total calcium burden in the coronary arteries; the CACS was quantified by a semiautomated computerized software program using the Agatston scoring method [13]. Based on the quantified CACS, coronary artery calcification in this study was classified as none to mild (a combination of CACS of 0 and CACS of 1 and 100), moderate (CACS of 101–400), or severe (CACS of >400) [14].

### 2.4. Post-Transplant Follow-Up

Post-transplant weight gain, diabetes, hypertension, and dyslipidemia may contribute to the development or progression of preexisting CVD in the post-transplant period [15], which can be managed using an immunosuppressive drug such as a calcineurin inhibitor (cyclosporine) [16]. In our center, calcineurin inhibitor-sparing strategies are employed in LT recipients for immunosuppressive treatment; mycophenolate mofetil or sirolimus is commonly used when the cyclosporine dose is reduced. Smoking and alcohol are strictly prohibited after LT. Patients at higher risk of developing major adverse cardiac events (MACEs) should be closely followed up and receive more aggressive medical management after transplantation. A multidisciplinary team, including a cardiologist and an endocrinologist, is assigned to manage the patients.

### 2.5. Data Acquisition and Outcome Measures

Considering the potential risk factors for MACEs, patients’ characteristics, preoperative medications and laboratory values, and intraoperative and postoperative variables were selected and reported after the detailed review of their electronic medical records. According to the American Heart Association (AHA)/American College of Cardiology Foundation (ACCF) scientific statement, the CAD risk factors for LT were diabetes mellitus, hypertension, history of cardiovascular disease, left-ventricular hypertrophy [LVH], age >60 years, smoking, and dyslipidemia [5]. LVH was defined as left-ventricular mass index values of >115 g/m^2^ for male patients and >95 g/m^2^ for female patients. Dyslipidemia was defined as the presence of any of the following abnormal lipid profiles: a total cholesterol level of ≥240 mg/dL, a low-density lipoprotein level of ≥190 mg/dL, a triglyceride level of ≥500 mg/dL, or high-density lipoprotein levels of <40 mg/dL for male patients and <50 mg/dL for female patients [11]. MACE and mortality information were also obtained. Follow-up data at 5 years after LDLT were censored. The primary outcome was to determine the CTCA findings that could be independently associated with MACEs within 5 years after LDLT. MACEs were assessed according to the following definitions: death from any cardiac cause, nonfatal myocardial infarction (MI), new-onset heart failure, cardiac arrest, supraventricular tachycardia requiring intervention, atrial fibrillation or flutter, symptomatic stable ventricular tachycardia requiring treatment, complete heart block, or stroke [17]. Data on all-cause mortality were collected, and the overall survival was analyzed according to the MACEs that the patient developed.

### 2.6. Statistical Analyses

Statistical analyses were performed using the R software version 3.5 (R Foundation for Statistical Computing, Vienna, Austria) and SPSS 21.0 software (IBM Corporation, Armonk, NY, USA). LT recipients were divided into the MACE group (recipients with MACEs within 5 years after LDLT) and non-MACE group (recipients without MACEs). For the descriptive analysis, variables were expressed as numbers (percentages), means (standard deviations), or medians (interquartile ranges) as appropriate. Categorical variables were compared using the chi-square or Fisher’s exact test, while continuous variables were compared using a t-test or Mann–Whitney U-test. For investigating the association between MACEs within 5 years after LDLT and predictor variables, Cox proportional hazards models were used. The noncardiac causes of death could be the competing risk events that triggered the occurrence of MACEs, including the cardiac causes of death; hence, a competing risk survival analysis was needed. Therefore, we constructed the Fine and Gray proportional subdistribution hazards model to predict MACEs within 5 years after LDLT considering the noncardiac cause of death as a competing event [18], which was estimated based on the subdistribution hazard ratios (HRs) and 95% confidence intervals (CIs). To assess the incremental prognostic value of the CTCA findings, these findings were categorized as follows: (1) a CACS of >400 plus more than 2-vessel disease, (2) CACS of >400 plus obstructive CAD, and (3) CACS of >400 plus mixed plaque. A CACS of >400 plus more than 1-vessel disease was not included as a category as its definition was identical to that of a CACS of >400 plus obstructive CAD. With regard to the clinical characteristics, laboratory values, preoperative evaluation results, and intraoperative variables, variables with a *p* value of <0.2 in the univariate analysis were presented. The inclusion of variables in the final multivariate Cox regression analysis to evaluate their prognostic value for MACEs within 5 years after LDLT was based on biological plausibility, clinical importance, and statistical considerations, which were limited by the number of covariates based on the number of clinical outcomes. The Kaplan–Meier methods with the log-rank test were used to compare the MACE-free survival rates between a CACS of >400 plus obstructive CAD and no CACS of >400 plus obstructive CAD reported. In addition, the overall survival was compared using the Kaplan–Meier methods according to the MACEs that the patient developed. A *p* value of <0.05 was considered significant.

## 3. Results

### 3.1. Transplant Recipient’s Characteristics

The clinical characteristics of LT recipients are shown in Table 1. In the cohort, 48 patients (7.6%) presented with MACEs within the follow-up period; of them, 79.9% were men with a median age of 53.0 (49.0–57.0) years. The main causes for requiring LT were hepatitis B virus-related liver cirrhosis (68.6%) and a median model for end-stage liver disease (MELD) score of 12.0 (9.0–17.0). Age was significantly different between the groups. In terms of the AHA/ACCF risk factors, significant difference was observed in the incidence of diabetes, hypertension, and dyslipidemia between the MACE and non-MACE groups. The Child–Turcotte–Pugh and MELD scores in the MACE group were significantly higher than those in the non-MACE group. Other variables did not differ.

### 3.2. Preoperative Evaluation Findings and Intraoperative Variables

Hemoglobin and high- and low-density lipoprotein cholesterol levels were significantly lower in the MACE group than in the non-MACE group (Table 2). By contrast, creatinine levels were significantly higher in the MACE group than in the non-MACE group (0.9 [0.7–1.5] vs. 0.7 [0.6–0.9], *p* < 0.001). The results of cardiac examinations, including echocardiography, corrected QT interval, and thallium SPECT, were not different between the two groups. During the intraoperative period, patients in the MACE group had significantly more red blood cell and fresh frozen plasma transfusions than those in the non-MACE group (Table 3). No significant differences were observed in the other intraoperative variables between the two groups.

### 3.3. CTCA Findings

Table 4 shows the preoperative CTCA findings in the transplant recipients. Among these recipients, severe coronary artery calcification (CACS of >400) was observed in 26 patients (4.1%). Calcified, noncalcified, and mixed plaques were observed in 150 (23.9%), 23 (3.7%), and 52 (8.3%) patients, respectively. Meanwhile, 36 (5.8%) patients had obstructive CAD. A total of 12 patients (3.5%) had 1-vessel CAD, 14 (2.1%) had 2-vessel CAD, and 1 (0.2%) had 3-vessel CAD. All CTCA findings were significantly different between the MACE and non-MACE groups (CACS, *p* < 0.001; coronary artery plaque, *p* = 0.04; coronary artery stenosis, *p* < 0.001; CAD, *p* < 0.001).

### 3.4. Long-Term Clinical Outcomes after LDLT According to the CTCA Findings and MACEs

**Figure 2:**
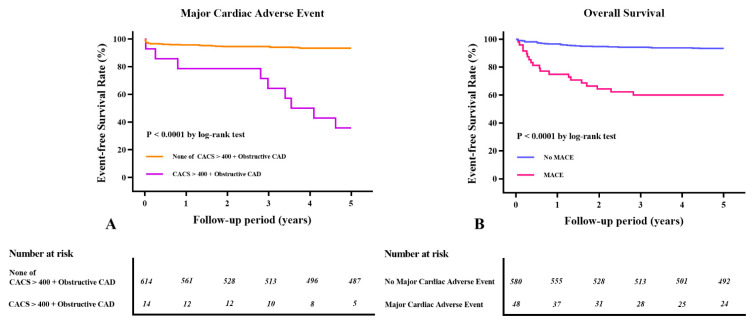
Kaplan–Meier curves. (**A**) Major adverse cardiac event (MACEs) according to the coronary artery calcium score (CACS) of >400 combined with obstructive coronary artery disease (CAD). (**B**) Overall survival according to the MACEs that developed.

During the follow-up period (within 5 years), 48 patients (7.6%) developed one or more MACEs. Of them, 7 patients had MI, 9 had new-onset heart failure, 24 had atrial fibrillation or flutter, 4 had a cardiac arrest, and 8 had a stroke. Four patients had both heart failure and atrial fibrillation. The Kaplan–Meier survival curves showed significant differences in the MACE-free survival rate among patients with a CACS of >400 plus obstructive CAD (log-rank *p* < 0.0001, Figure 2A). The primary causes of perioperative mortality were recurrence of hepatocellular carcinoma (28.6%), nonhepatic infectious diseases, including pneumonia and sepsis (23.2%), graft failure (23.2%), MACEs (5.4%), and other causes (19.6%) such as bleeding, nonhepatocellular carcinoma, and diseases with unknown origins. Fifty-six patients (8.9%) died within 5 years after LDLT. The 5-year survival rates of the MACE and non-MACE groups were 60.4% and 93.6%, respectively. The Kaplan–Meier survival curves showed significant differences in the overall mortality rate according to the MACEs that developed (log-rank: *p* < 0.0001, Figure 2B).

### 3.5. Prognostic Value of CTCA for Long-Term MACE

**Figure 3:**
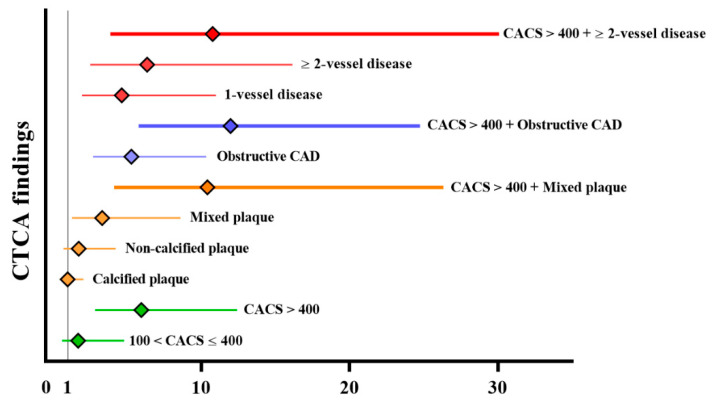
Unadjusted hazard ratio of the CTCA findings for predicting long-term major adverse cardiac events. CACS, coronary artery calcium score; CAD, coronary artery disease; diamond, hazard ratio; horizontal line, 95% confidence interval.

The results of an unadjusted Cox regression analysis of long-term MACEs are presented in Figure 3. Mixed plaque, obstructive CAD, 1- and ≥2-vessel CAD, and a CACS of >400 were significantly associated with MACEs within 5 years after LDLT (HR, 3.31 [95% CI: 1.28–8.60], *p* = 0.014; 4.64 [1.96–10.99], *p* < 0.001; 6.35 [2.50–16.16], *p* = 0.001; 5.289 [2.70–10.37], *p* < 0.001; 5.96 [2.84–12.41], *p* < 0.001; Figure 3). Considering that a CACS of >400 was a predictor of early cardiovascular complications in our previous study [2], each variable was combined with a CACS of >400. The incremental value of adding CTCA with a CACS of >400 is demonstrated in Figure 3. The HR of CACS of >400 combined with obstructive CAD was the highest in the composites of CTCA findings, which was significantly associated with MACEs within 5 years after LDLT (11.97 [5.79–24.75], *p* < 0.001). Table 5 shows the Cox proportional and Fine and Gray proportional subdistribution hazards models for MACEs within 5 years after LDLT. The crude and multivariable HRs and 95% CIs of the variables were similar between the two regression models. The Fine and Gray’s subdistribution hazards model revealed that age, diabetes, dyslipidemia, creatinine, and a CACS of >400 combined with obstructive CAD were independently associated with MACEs within 5 years after LDLT (1.05 [1.01–1.10], *p* = 0.018; 2.43 [1.37–4.29], *p* = 0.002; 2.45 [1.23–4.70], *p* = 0.023; 1.19 [1.08–1.3], *p* = 0.010; 5.01 [2.37–10.58], *p* < 0.001; Table 5).

## 4. Discussion

Our main finding was that a CACS of >400 combined with obstructive CAD was significantly associated with MACEs within 5 years after LDLT. Because LT candidates have a high risk of CAD and cardiovascular complications occur frequently after LT, preoperative CTCA is necessary as an accurate noninvasive tool for screening CAD before LT [19,20,21,22]. Previous studies on CTCA in LT recipients have emphasized the individual prognostic values of CACS and CTCA for early cardiovascular complications [2,11]. To the best of our knowledge, this is the first study to investigate the association between CTCA in combination with CACS and long-term MACEs after LDLT. Furthermore, the results of this study expand the findings of our previous CTCA study conducted in LT recipients, which investigated the advantage of CACS in risk assessment.

In several meta-analyses, a higher CACS has been associated with a greater degree of coronary artery stenosis and a higher risk of coronary heart disease [23,24]. Of note, a CACS of >400 is significantly associated with coronary artery stenosis on coronary angiography in asymptomatic patients and LT candidates [10]. In addition, CACS is known to be more sensitive in detecting the cardiovascular risk factors in LT patients than the Framingham risk score [8]. In patients with either chronic renal failure or asymptomatic diabetes, a CACS of >400 was a prognostic indicator of cardiovascular events [25,26]. In our previous study, we demonstrated that a CACS of >400 and female sex are reliable predictive factors for early postoperative cardiovascular complications after LT [2]. Subsequently, when we analyzed the long-term MACEs after LDLT in this study, a CACS of >400 still provided prognostic values. The unadjusted HR for CACS of >400 is 5.96, which is better than that for 1-vessel disease (4.64) and lower than that for >2-vessel disease (6.35). However, traditional CAC scores make several assumptions about the biology of calcification and atherosclerosis and fail to capture information about the regional distribution of calcification within the coronary tree. Further, none of the scores incorporate information on the number or size of calcified coronary lesions [27]. Therefore, the clinical implication of CAC scores can be limited, and the interpretations of CACS combined with CTCA are emphasized.

CTCA is validated as a potential alternative to coronary angiography for diagnosing and grading the severity of CAD [28]. Preoperative CTCA is predictive of MACE during the 1 year follow-up period after LT [29]. Recently, Moon et al. [11] described the relationship between CTCA-diagnosed CAD and post-LT MI based on the measured postoperative troponins within 1 month after LT at our center. They found that the increasing severity of CTCA-diagnosed CAD results in the high prevalence of early post-LT MI. In this study, CTCA-diagnosed CAD and mixed plaque were significantly related to long-term MACEs after LDLT. These results implicate that CTCA-diagnosed CAD may have a prognostic value for both short-term and long-term CVD. In addition, recent scientific evidence shows that CTCA allows the early detection of CAD and adverse plaque characteristic, diagnosis of significant stenosis, and determination of appropriate treatment for the prevention of adverse cardiovascular outcomes after liver transplantation [30].

Considering the significant diagnostic and prognostic values of CTCA, including CACS, further investigations on the advantages of CTCA in combination with the CACS are warranted. Hou et al. [31] showed that CACS combined with the CTCA findings had an incremental prognostic value over routine risk factors for long-term MACE in 5007 outpatients who were suspected of having CAD. Although CTCA is superior to CACS and traditional risk factors, CTCA in combination with CACS has a significantly improved predictive value for MACEs. Our results are consistent with those of a previous study. The association between CTCA and long-term MACEs is slightly inferior to that of CACS and long-term MACEs; after CTCA combined with CACS, its association has increased nearly twice. In the multivariate Cox regression, a CACS of >400 in combination with obstructive CAD is independently associated with long-term MACEs after LDLT. In the study by Kwon et al. [32], CACS does not add a prognostic value to standard CTCA in low-risk patients suspected of having CAD. However, transplant recipients are at a high risk of CAD [19]. Therefore, CTCA in combination with CACS appears to provide an incremental prognostic value for long-term MACEs in high-risk patients suspected of having CAD based on our current results and previous study findings. Importantly, although several perioperative variables or findings associated with early postoperative cardiovascular complications after LT were surveyed [2,11,33], the factors related to long-term MACEs (within 5 years) have been rarely investigated. To the best of our knowledge, this is the first study to demonstrate the incremental prognostic value of CTCA in combination with CACS for the prediction of long-term cardiovascular complications after LDLT.

According to a previous study conducted in American populations [4], CVD developed in 20.7% of recipients within 5 years after LT. Compared with this study, the incidence of CVD within 5 years after LDLT in the South Korean population was relatively lower (7.6%) in our study, which could be partially explained by the lower rate of postoperative complications after LDLT [34]. Despite the low rate of MACEs in this study, a CACS of >400 combined with obstructive CAD showed a significant prognostic value for long-term MACEs in LT recipients. Thus, it could be applicable to other populations (North Americans or Europeans) and to orthotopic LT patients with a high rate of MACEs, although further external validation is needed.

Given the limited evaluating tool for preoperative cardiac risk stratification in LT recipients [35] and low performance of noninvasive stress testing such as myocardial perfusion scans or dobutamine stress echocardiography [5,36], we suggest that CTCA may be considered as an alternative initial screening test for risk stratification in LT recipients, as it can determine preoperatively the presence and extent of CAD and identify the high-risk patients with early or late CVD after LT.

In this study, age, diabetes, dyslipidemia, and creatinine were independently associated with long-term MACEs after LDLT, which is consistent with the findings of previous studies [4,37]. Our study showed that a CACS of >400 combined with obstructive CAD might be superior to conventional coronary risk factors in terms of predicting long-term MACEs.

This study has several strengths. Previous studies reporting the predictive factors for postoperative cardiovascular complications are limited by a small sample size or sampling bias because cardiac evaluation is often performed in patients with either known CVD or those with known high-risk factors [16,38]. In addition, as several of the accepted risk factors for CVD, such as hyperlipidemia, hypertension, and diabetes, are often affected by the physiological changes that occur in patients with end-stage liver disease, alternative markers for an increased CVD risk in those with no known CVD are needed [4]. Therefore, to strengthen the association between CTCA in combination with CACS and long-term MACEs after LDLT, we excluded patients with CVD in this study. In this study, the incidence of MACE-related death was 5.4%, which was relatively lower than that reported by previous studies (8.3–12.1%) [16,39]. This may be due to the exclusion of the recipients with CVD in the analysis and may represent an accurate MACE-related death rate after LT, excluding deaths caused by underlying CVD.

This study has some limitations. First, the definition of a postoperative cardiovascular complication such as a MACE varies in previous studies; thus, the outcomes of this study may change according to the definition used. However, we defined cardiovascular complication as a MACE, which is a composite endpoint frequently used in cardiovascular research and for postoperative CVD after LT [17,40]. Second, some studies found that immunosuppressive drugs played an important role in the incidence of late CVD [16,41]. Steroid-free and/or immunosuppressive regimens including mycophenolate mofetil may be associated with lower cardiovascular morbidity and mortality [16]. In addition, tacrolimus may be associated with decreasing the risk of de novo arterial hypertension and dyslipidemia compared to cyclosporine. Although the protocol of calcineurin inhibitor-sparing strategies was applied at our institution to minimize metabolic disturbance and occurrence of CVD, this study did not evaluate the effect of immunosuppressive drugs on long-term MACEs, resulting in a major limitation of this study. Third, data collection was limited due to the retrospective nature of the study. To improve the quality of data, we excluded patients with incomplete data. Fifth, the South Korean liver transplant population in this study is distinct from other populations (such as those from North America or Europe), especially in terms of the prevalence of LDLT, nonalcoholic steatohepatitis, and MACEs. Therefore, the results of our study should be interpretated with caution and must be externally validated.

## 5. Conclusions

Age, diabetes, dyslipidemia, and creatinine levels may be independently associated with long-term MACEs after LDLT. Importantly, obstructive CAD on CTCA in combination with a CACS of >400 may be associated with an incremental prognostic value compared with other predictive factors. These findings suggest that preoperative CTCA in combination with CACS appears to be a promising noninvasive modality with a significant prognostic value for long-term MACEs in transplant recipients. However, further external validation is needed.

## Figures and Tables

**Table 1:** Clinical characteristics of liver transplant recipients according to the major adverse cardiac events that developed.

Variables	Total (*n* = 628)	Non-MACE (*n* = 580)	MACE (*n* = 48)	*p* Value
Age (years)	53.0 (8.0)	52.0 (9.0)	54.0 (8.5)	0.006
Sex (male)	502 (79.9)	462 (79.7)	40 (83.3)	0.672
Body mass index (kg/m^2^)	23.5 (4.5)	23.5 (4.5)	22.6 (4.4)	0.205
AHA/ACCF risk factors for CAD				
Age > 60 years	74 (11.8)	64 (11.0)	10 (20.8)	0.073
Diabetes mellitus	144 (22.9)	123 (21.2)	21 (43.8)	0.001
Hypertension	81 (12.9)	69 (11.9)	12 (25.0)	0.017
History of cardiovascular disease	0 (0)	0 (0)	0 (0)	
Dyslipidemia	307 (52.9)	344 (54.8)	37 (77.1)	0.002
Current smoker	118 (18.8)	109 (18.8)	9 (18.8)	0.999
Left-ventricular hypertrophy	63 (10.0)	59 (10.2)	4 (9.3)	0.875
Preoperative medication				
ACE inhibitor	31 (4.9)	27 (4.7)	4 (8.3)	0.433
Beta-blocker	160 (25.5)	144 (24.8)	16 (33.3)	0.260
Diuretics	246 (39.2)	222 (38.3)	24 (50.0)	0.148
Statin	11 (1.8)	8 (1.4)	3 (6.2)	0.057
Alcohol history	171 (27.2)	152 (26.2)	19 (38.8)	0.122
Underlying liver diseases				0.596
Hepatitis B virus	431 (68.6)	399 (68.8)	32 (66.7)	
Hepatitis C virus	51 (8.1)	45 (7.8)	6 (12.5)	
Non-B non-C hepatitis	19 (3.0)	18 (3.1)	1 (2.1)	
Alcoholic liver disease	75 (11.9)	68 (11.7)	7 (14.6)	
Others	52 (8.3)	50 (8.6)	2 (4.2)	
Child–Turcotte–Pugh score	8.0 (4.0)	8.0 (3.0)	8.5 (3.0)	0.045
Model for end-stage liver disease score	12.0 (8.0)	12.0 (8.0)	14.5 (10.5)	0.025

Values are expressed as medians (interquartile ranges) for continuous variables and numbers (percentages) for categorical variables. MACE, major adverse cardiac events; AHA/ACCF, American Heart Association/American College of Cardiology Foundation; CAD, coronary artery disease; ACE inhibitor, angiotensin-converting enzyme inhibitor. Others include toxic and autoimmune hepatitis, Budd–Chiari syndrome, primary biliary cirrhosis, and Wilson’s disease.

**Table 2:** Preoperative evaluation findings of liver transplant recipients according to the major adverse cardiac events that developed.

Variables	Total (*n* = 628)	Non-MACE (*n* = 580)	MACE (*n* = 48)	*p* Value
Hemoglobin (g/dL)	10.9 (3.4)	11.0 (3.4)	9.9 (3.5)	0.021
Platelet (10^3^/mm)	58.0 (46.0)	58.0 (46.5)	61.0 (45)	0.277
Prothrombin time (INR)	1.4 (0.4)	1.4 (0.4)	1.4 (0.4)	0.510
Albumin (g/dL)	3.2 (0.9)	3.2 (0.9)	3.0 (0.9)	0.186
Creatinine (mg/dL)	0.7 (0.3)	0.7 (0.3)	0.9 (0.8)	<0.001
Total cholesterol (mg/dL)	117.5 (59.5)	120.0 (58.0)	99.5 (69.5)	0.023
HDL cholesterol (mg/dL)	39.0 (24.0)	40.0 (25.0)	32.0 (20.0)	0.004
LDL cholesterol (mg/dL)	64.0 (42.0)	65.0 (41.0)	48.0 (53.5)	0.044
Triglyceride (mg/dL)	61.0 (38.0)	60.0 (39.0)	65.0 (34.0)	0.223
Echocardiography				0.315
Valve diseases	23 (3.7)	21 (3.6)	2 (4.2)	
Pulmonary hypertension	9 (1.4)	8 (1.4)	1 (2.1)	
Other abnormalities	6 (0.9)	4 (0.9)	2 (4.2)	
QTc (ms)	446.0 (40.0)	446.0 (39.0)	449.5 (49.0)	0.305
Abnormal thallium SPECT	6 (1.0)	5 (0.9)	1 (2.5)	0.881

Values are expressed as medians (interquartile ranges) for continuous variables and numbers (percentages) for categorical variables. INR, international normalized ratio; HDL, high-density lipoprotein; LDL, low-density lipoprotein; QTc, corrected QT interval. Other abnormalities in echocardiography include pericardial effusion, wall motion abnormality, and septal defect.

**Table 3:** Intraoperative variables of liver transplant recipients according to the major adverse cardiac events that developed.

Variables	Total (*n* = 628)	Non-MACE (*n* = 580)	MACE (*n* = 48)	*p* Value
Surgical time (min)	787.0 (135.0)	787.0 (135)	786.0 (137.5)	0.698
RBC transfusion (unit)	7.0 (11.0)	7.0 (11.0)	12.0 (10.5)	0.002
FFP transfusion (unit)	10.0 (12.0)	9.0 (11.0)	12.0 (14.5)	0.003
Cryoprecipitate transfusion (unit)	10.0 (10.0)	10.0 (10.0)	10.0 (17.5)	0.728
Apheresis platelet transfusion (unit)	1.0 (2.0)	1.0 (2.0)	1.0 (2.0)	0.243
Graft volume (g)	730.0 (180.0)	730.0 (175)	750.0 (202.5)	0.411
Graft-recipient weight ratio (%)	1.1 (0.4)	1.1 (0.4)	1.1 (0.3)	0.369
Ischemic time (min)	124.0 (37.0)	123.5 (35.5)	130.0 (47.0)	0.460

Values are expressed as medians (interquartile ranges) for continuous variables. RBC, red blood cell; FFP, fresh-frozen plasma.

**Table 4:** Preoperative findings of the coronary computed tomographic angiography of liver transplant recipients according to the major adverse cardiac events that developed.

Variables	Total (*n* = 628)	Non-MACE (*n* = 580)	MACE (*n* = 48)	*p* Value
Coronary artery calcium score				<0.001
None−mild (≤100)	563 (89.6)	528 (91.0)	35 (72.9)	
Moderate (101−400)	39 (6.2)	35 (6.0)	4 (8.3)	
Severe (>400)	26 (4.1)	17 (2.9)	9 (18.8)	
Coronary artery plaque				0.040
Nonplaque	403 (64.2)	376 (64.8)	27 (56.2)	
Calcified plaque	150 (23.9)	140 (24.1)	10 (20.8)	
Noncalcified plaque	23 (3.7)	18 (3.1)	5 (10.4)	
Mixed plaque	52 (8.3)	46 (7.9)	6 (12.5)	
Coronary artery stenosis				<0.001
Nonobstructive (<50%)	592 (94.3)	555 (95.7)	37 (77.1)	
Obstructive (≥50%)	36 (5.8)	25 (4.3)	11 (22.9)	
Coronary artery diseases				<0.001
Noncoronary artery disease	592 (94.3)	555 (95.7)	37 (77.1)	
1-vessel disease	21 (3.5)	15 (2.6)	6 (12.5)	
2-vessel diseases	14 (2.1)	10 (1.7)	4 (8.3)	
3-vessel diseases	1 (0.2)	0 (0.0)	1 (2.1)	

Values are expressed as numbers (percentages) for categorical variables.

**Table 5:** Cox proportional and the Fine and Gray proportional subdistribution hazards models for major adverse cardiac events within 5 years after living donor liver transplantation.

Variables	Cox Proportional Hazards Model				Fine and Gray’s Subdistribution Hazards Model			
	Crude HR (95% CI)	*p* Value	Multivariable HR(95% CI)	*p* Value	Crude sHR (95% CI)	*p* Value	Multivariable sHR (95% CI)	*p* Value
Age	1.07 (1.02–1.12)	0.002	1.05 (1.01–1.10)	0.018	1.07 (1.03–1.11)	0.001	1.05 (1.01–1.10)	0.018
Diabetes mellitus	2.72 (1.54–4.81)	0.001	2.22 (1.24–3.99)	0.007	2.74 (1.55–4.84)	<0.001	2.43 (1.37–4.29)	0.002
Hypertension	2.27 (1.18–4.36)	0.014			2.29 (1.21–4.33)	0.011		
Dyslipidemia	2.90 (1.48–5.69)	0.002	2.27 (1.14–4.51)	0.019	2.92 (1.52–5.70)	0.003	2.45 (1.23–4.70)	0.023
Statin therapy	4.03 (1.25–12.97)	0.019			3.94 (1.28–12.08)	0.017		
Diuretics	0.80 (0.60–1.06)	0.112			1.58 (0.90–2.78)	0.112		
MELD score	1.05 (1.01–1.08)	0.006			1.05 (1.01–1.08)	0.008		
CTP score	1.12 (0.99–1.26)	0.071			1.11 (1.00–1.24)	0.052		
Hemoglobin	0.86 (0.76–0.99)	0.029			0.86 (0.74–1.00)	0.054		
Albumin	0.70 (0.44–1.13)	0.151			0.71 (0.43–1.18)	0.189		
Creatinine	1.26 (1.14–1.39)	<0.001	1.20 (1.06–1.36)	0.003	1.25 (1.12–1.41)	<0.001	1.19 (1.08–1.3)	<0.001
Total cholesterol	1.01 (0.99–1.01)	0.034			0.99 (0.99–1.00)	0.058		
HDL cholesterol	0.98 (0.97–0.99)	0.016			0.98 (0.96–0.99)	0.009		
LDL cholesterol	0.99 (0.98–1.00)	0.067			0.99 (0.98–1.00)	0.072		
RBC transfusion	1.02 (1.01–1.03)	0.017			1.02 (1.00–1.03)	0.005		
FFP transfusion	1.02 (1.01–1.03)	0.031			1.01 (1.00–1.03)	0.009		
Abnormal echocardiography	1.85 (0.73–4.67)	0.194			1.90 (0.76–4.74)	0.172		
CACS > 400 + obstructive CAD	11.97 (5.79–24.75)	<0.001	5.02 (2.25–11.21)	<0.001	11.70 (6.42~21.32)	<0.001	5.01 (2.37–10.58)	<0.001

HR, hazard ratio; CI, confidence interval; sHR, subdistribution hazard ratio; MELD, model for end-stage liver disease; CTP, Child–Turcotte–Pugh; HDL, high-density lipoprotein; LDL, low-density lipoprotein; RBC, red blood cell transfusions; FFP, fresh-frozen plasma; CACS, coronary artery calcium score.

## Data Availability

The datasets generated during and/or analyzed during the current study are available from the corresponding author upon reasonable request.

## References

[1] VanWagner L.B., Lapin B., Levitsky J., Wilkins J.T., Abecassis M.M., Skaro A.I., Lloyd-Jones D.M. (2014). High early cardiovascular mortality after liver transplantation. Liver Transpl..

[2] Kong Y.G., Kang J.W., Kim Y.K., Seo H., Lim T.H., Hwang S., Hwang G.S., Lee S.G. (2015). Preoperative coronary calcium score is predictive of early postoperative cardiovascular complications in liver transplant recipients. Br. J. Anaesth..

[3] Kong Y.G., Ha T.Y., Kang J.W., Hwang S., Lee S.G., Kim Y.K. (2015). Incidence and predictors of increased coronary calcium scores in liver transplant recipients. Transplant Proc..

[4] Fussner L.A., Heimbach J.K., Fan C., Dierkhising R., Coss E., Leise M.D., Watt K.D. (2015). Cardiovascular disease after liver transplantation: When, what, and who is at risk. Liver. Transpl..

[5] Lentine K.L., Costa S.P., Weir M.R., Robb J.F., Fleisher L.A., Kasiske B.L., Carithers R.L., Ragosta M., Bolton K., Auerbach A.D. (2012). Cardiac disease evaluation and management among kidney and liver transplantation candidates: A scientific statement from the American Heart Association and the American College of Cardiology Foundation. J. Am. Coll. Cardiol..

[6] Snipelisky D., Levy M., Shapiro B. (2014). Utility of dobutamine stress echocardiography as part of the pre-liver transplant evaluation: An evaluation of its efficacy. Clin. Cardiol..

[7] Mowatt G., Cook J.A., Hillis G.S., Walker S., Fraser C., Jia X., Waugh N. (2008). 64-Slice computed tomography angiography in the diagnosis and assessment of coronary artery disease: Systematic review and meta-analysis. Heart.

[8] Choi J.M., Kong Y.G., Kang J.W., Kim Y.K. (2017). Coronary computed tomography angiography in combination with coronary artery calcium scoring for the preoperative cardiac evaluation of liver transplant recipients. Biomed. Res. Int..

[9] McAvoy N.C., Kochar N., McKillop G., Newby D.E., Hayes P.C. (2008). Prevalence of coronary artery calcification in patients undergoing assessment for orthotopic liver transplantation. Liver Transpl..

[10] Kemmer N., Case J., Chandna S., Neff G.W. (2014). The role of coronary calcium score in the risk assessment of liver transplant candidates. Transplant Proc..

[11] Moon Y.J., Kwon H.M., Jung K.W., Jeong H.W., Park Y.S., Jun I.G., Song J.G., Hwang G.S. (2019). Risk stratification of myocardial injury after liver transplantation in patients with computed tomographic coronary angiography-diagnosed coronary artery disease. Am. J. Transplant..

[12] Pohle K., Achenbach S., Macneill B., Ropers D., Ferencik M., Moselewski F., Hoffmann U., Brady T.J., Jang I.K., Daniel W.G. (2007). Characterization of non-calcified coronary atherosclerotic plaque by multi-detector row CT: Comparison to IVUS. Atherosclerosis.

[13] Agatston A.S., Janowitz W.R., Hildner F.J., Zusmer N.R., Viamonte M., Detrano R. (1990). Quantification of coronary artery calcium using ultrafast computed tomography. J. Am. Coll. Cardiol..

[14] Erbel R., Mohlenkamp S., Moebus S., Schmermund A., Lehmann N., Stang A., Dragano N., Gronemeyer D., Seibel R., Kalsch H. (2010). Coronary risk stratification, discrimination, and reclassification improvement based on quantification of subclinical coronary atherosclerosis: The Heinz Nixdorf Recall study. J. Am. Coll. Cardiol..

[15] Luca L., Westbrook R., Tsochatzis E.A. (2015). Metabolic and cardiovascular complications in the liver transplant recipient. Ann. Gastroenterol..

[16] D’Avola D., Cuervas-Mons V., Martí J., Ortiz de Urbina J., Lladó L., Jimenez C., Otero E., Suarez F., Rodrigo J.M., Gómez M.A. (2017). Cardiovascular morbidity and mortality after liver transplantation: The protective role of mycophenolate mofetil. Liver Transpl..

[17] Vanwagner L.B., Bhave M., Te H.S., Feinglass J., Alvarez L., Rinella M.E. (2012). Patients transplanted for nonalcoholic steatohepatitis are at increased risk for postoperative cardiovascular events. Hepatology.

[18] Austin P.C., Fine J.P. (2017). Practical recommendations for reporting Fine-Gray model analyses for competing risk data. Stat. Med..

[19] Mandell M.S., Lindenfeld J., Tsou M.Y., Zimmerman M. (2008). Cardiac evaluation of liver transplant candidates. World J. Gastroenterol..

[20] Therapondos G., Flapan A.D., Plevris J.N., Hayes P.C. (2004). Cardiac morbidity and mortality related to orthotopic liver transplantation. Liver Transpl..

[21] An J., Shim J.H., Kim S.O., Lee D., Kim K.M., Lim Y.S., Lee H.C., Chung Y.H., Lee Y.S. (2014). Prevalence and prediction of coronary artery disease in patients with liver cirrhosis: A registry-based matched case-control study. Circulation.

[22] Di Carli M.F., Blankstein R. (2014). Low yield of routine preoperative coronary computed tomography angiography in patients evaluated for liver transplantation. Circulation.

[23] Shaw L.J., Raggi P., Schisterman E., Berman D.S., Callister T.Q. (2003). Prognostic value of cardiac risk factors and coronary artery calcium screening for all-cause mortality. Radiology.

[24] Pletcher M.J., Tice J.A., Pignone M., Browner W.S. (2004). Using the coronary artery calcium score to predict coronary heart disease events: A systematic review and meta-analysis. Arch. Intern. Med..

[25] Rosário M.A., Lima J.J., Parga J.R., Avila L.F., Gowdak L.H., Lemos P.A., Rochitte C.E. (2010). Coronary calcium score as predictor of stenosis and events in pretransplant renal chronic failure. Arq. Bras. Cardiol..

[26] Jeevarethinam A., Venuraju S., Dumo A., Ruano S., Atwal S., Mehta V.S., Rakhit R., Lahiri A. (2014). Relationship between coronary artery calcification (CAC) and carotid atherosclerosis in asymptomatic diabetes: A prospective study. Heart.

[27] Michael J., Martin B., Sina K., Rajesh T.-M., Miguel C.-A. (2017). Coronary artery calcium scoring: Is it time for a change in methodology?. JACC Cardiovasc. Imaging.

[28] Abdulla J., Abildstrom S.Z., Gotzsche O., Christensen E., Kober L., Torp-Pedersen C. (2007). 64-multislice detector computed tomography coronary angiography as potential alternative to conventional coronary angiography: A systematic review and meta-analysis. Eur. Heart J..

[29] Cassagneau P., Jacquier A., Giorgi R., Amabile N., Gaubert J.Y., Cohen F., Muller C., Jolibert M., Louis G., Varoquaux A. (2012). Prognostic value of preoperative coronary computed tomography angiography in patients treated by orthotopic liver transplantation. Eur. J. Gastroenterol. Hepatol..

[30] Steinkohl F., Barbieri F., Senoner T., Strobl S., Finkenstedt A., Plank F., Langer C., Beyer C., Birkl K., Widmann G. (2021). Coronary atherosclerosis profile in patients with end-stage liver disease prior to liver transplantation due to alcoholic fatty liver: A coronary CTA study. Eur. Radiol..

[31] Hou Z.H., Lu B., Gao Y., Jiang S.L., Wang Y., Li W., Budoff M.J. (2012). Prognostic value of coronary CT angiography and calcium score for major adverse cardiac events in outpatients. JACC Cardiovasc. Imaging.

[32] Kwon S.W., Kim Y.J., Shim J., Sung J.M., Han M.E., Kang D.W., Kim J.Y., Choi B.W., Chang H.J. (2011). Coronary artery calcium scoring does not add prognostic value to standard 64-section CT angiography protocol in low-risk patients suspected of having coronary artery disease. Radiology.

[33] Jodocy D., Abbrederis S., Graziadei I.W., Vogel W., Pachinger O., Feuchtner G.M., Jaschke W., Friedrich G. (2012). Coronary computer tomographic angiography for preoperative risk stratification in patients undergoing liver transplantation. Eur. J. Radiol..

[34] Trevor W.R., Helena K., Tomohiro T., Paul D.G., Ian D.M., Mark S.C., Eberhard L.R., Markus S., Anand G., Gary L. (2013). Living donor versus deceased donor liver transplantation: A surgeon-matched comparison of recipient morbidity and outcomes. Transpl. Int..

[35] Hogan B.J., Gonsalkorala E., Heneghan M.A. (2017). Evaluation of coronary artery disease in potential liver transplant recipients. Liver Transpl..

[36] Davidson C.J., Gheorghiade M., Flaherty J.D., Elliot M.D., Reddy S.P., Wang N.C., Sundaram S.A., Flamm S.L., Blei A.T., Abecassis M.I. (2002). Predictive value of stress myocardial perfusion imaging in liver transplant candidates. Am. J. Cardiol..

[37] Haywood S., Abecassis M., Levitsky J. (2011). The renal benefit of mycophenolate mofetil after liver transplantation. Clin. Transplant..

[38] Wray C., Scovotti J.C., Tobis J., Niemann C.U., Planinsic R., Walia A., Findlay J., Wagener G., Cywinski J.B., Markovic D. (2013). Liver transplantation outcome in patients with angiographically proven coronary artery disease: A multi-institutional study. Am. J. Transplant..

[39] Jain A., Reyes J., Kashyap R., Dodson S.F., Demetris A.J., Ruppert K., Abu-Elmagd K., Marsh W., Madariaga J., Mazariegos G. (2000). Long-term survival after liver transplantation in 4,000 consecutive patients at a single center. Ann. Surg..

[40] VanWagner L.B., Serper M., Kang R., Levitsky J., Hohmann S., Abecassis M., Skaro A., Lloyd-Jones D.M. (2016). Factors associated with major adverse cardiovascular events after liver transplantation among a national sample. Am. J. Transplant..

[41] Rabkin J.M., Corless C.L., Rosen H.R., Olyaei A.J. (2002). Immunosuppression impact on long-term cardiovascular complications after liver transplantation. Am. J. Surg..

